# Tree‐Ring and Sediment Analyses Reveal Processes of Bald Cypress Ghost Forest Formation From Dredging in the Cape Fear River, North Carolina, USA

**DOI:** 10.1002/ece3.71677

**Published:** 2025-08-28

**Authors:** Kendra Devereux, Monica Rother, Andrea D. Hawkes, Philip Bresnahan, D. Reide Corbett, Roger Shew

**Affiliations:** ^1^ Department of Earth and Ocean Sciences University of North Carolina Wilmington Wilmington North Carolina USA; ^2^ Center for Marine Science University of North Carolina Wilmington Wilmington North Carolina USA; ^3^ Department of Environmental Sciences University of North Carolina Wilmington Wilmington North Carolina USA; ^4^ Department of Coastal Studies East Carolina University Greenville North Carolina USA

## Abstract

Forested, freshwater tidal wetlands in the southeastern US are dominated by bald cypresses ( *Taxodium distichum* ), which tolerate low levels of salinity. However, the response of old‐growth bald cypress trees to prolonged increases in salinity remains uncertain. Bald cypress ghost forests occur along Smith Creek, a tributary of the Cape Fear River, North Carolina which has been dredged multiple times since 1871. Atop relative sea‐level rise, dredging may be accelerating saltwater intrusion and ghost forest formation. To examine the effects of increased salinity on bald cypress and improve understanding of the process of ghost forest formation, we sampled trees and sediments along a salinity gradient in Smith Creek. We developed tree‐ring chronologies for a high‐salinity (8–12 ppt) and low‐salinity (< 5 ppt) site. We quantified growth suppression patterns and climate–growth relationships at each site. Agglutinated benthic foraminifera and thecamoebians found in sediment samples served as proxies for increasing salinity over time. At the high salinity site, foraminifera began to appear at 25 cm depth in the sediment profile (indicating elevated salinity since c.1950). A lack of foraminifera at the low‐salinity site indicated no major salinity increase. We identified five growth suppression events in bald cypress at the high‐salinity site that were not observed at the low‐salinity site (1859–1860, 1877–1887, 1946–1960, 1971–1983, 1985–2022), three of which are consistent with stress following years of dredging in the Cape Fear River (1946, 1970, 2000). The length and severity of the most recent suppression event at the high‐salinity site suggests that those bald cypress are experiencing permanently stressful conditions. Additionally, monthly correlation analyses indicated that these trees may have increased sensitivity to drought and temperature. Saltwater intrusion into forested freshwater tidal wetlands induces stress in bald cypress, facilitating ghost forest formation and the loss of important ecosystem services.

## Graphical abstract

In this study, we used a novel approach to combine the use of tree‐ring and pollution chronologies along with microfossil analysis to characterize saltwater intrusion and its influence on the process of ghost forest formation in a bald cypress wetland. We demonstrate suppressed growth and changes to climate–growth relationships in bald cypress chronologies in response to elevated salinity. Our findings highlight concerns for the persistence of old‐growth tidal bald cypress communities and their associated ecosystem services in the face of saltwater intrusion.

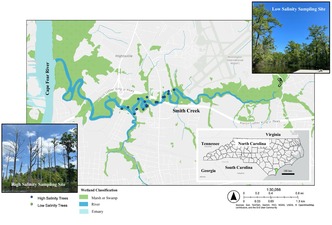

## 1 Introduction

Forested freshwater tidal wetlands dominated by bald cypress ( *Taxodium distichum* ) are common throughout the southeastern United States, occurring between forested uplands and lower tidal marshes (Field et al. 1991). These environments provide important ecosystem services and host a unique array of species, but due to their proximity to the coast, tidal influence, and transitional nature, they are vulnerable to impacts of saltwater intrusion (Field et al. 1991). When salinity increases, forested freshwater tidal wetlands can be converted to emergent marsh and ghost forests (i.e., standing dead trees in former forests; Kirwan and Gedan 2019; Schieder and Kirwan 2019). Subsequently, plant diversity decreases, and ecosystem services can be lost (Ury et al. 2021; Hackney et al. 2007; Odum et al. 1984). In most locations, the dominant mechanism for saltwater intrusion in forested freshwater tidal wetlands is global sea‐level rise (+0.20 m since 1900; Fox‐Kemper et al. 2021). However, local and regional factors along coasts also cause variations in the amount and rate of sea‐level rise in a given area, which is referred to as relative sea‐level rise (RSLR; Nicholls et al. 2021).

The Cape Fear River, in southeastern North Carolina, USA, has been dredged regularly to maintain depths suitable for navigational purposes (Table 1; US Army Corps of Engineers 2022). Since 1871, the U.S. Army Corps of Engineers has conducted 10 major dredging projects, deepening the Wilmington Harbor over a length of ~35 km (21.7 miles) from less than ~3.66 m (12 ft) below Mean Lower Low Water (MLLW) to its current depth of 12.80 m (42 ft) below MLLW (Table 1; Leonard et al. 2011). Consistent maintenance dredging keeps the Harbor at a 12.80 m depth. Familkhalili and Talke (2016) found that the tidal amplitude at the Wilmington tide gauge (operated by the National Oceanographic and Atmospheric Administration, NOAA) nearly doubled from ~0.35 m in the late nineteenth/early twentieth century to ~0.65 m in 2015 because of channel deepening that led to reduced hydraulic resistance. Combined with higher mean water levels caused by RSLR, greater tidal amplitude has carried saltwater farther up the Cape Fear River than would occur in an unmodified river channel. Many freshwater tidal swamps that existed along the Cape Fear River and its tributaries, including Smith Creek, have been converted to brackish and saline emergent wetlands with ghost forests due to these dredging effects (Magolan and Halls 2020).

**TABLE 1:** Years and depths below MLLW of major dredging events in Wilmington Harbor (modified from Leonard et al. 2011).

Year	Wilmington Harbor dredge depth
1871	3.66 m
1881	4.88 m
1890	6.10 m
1909	7.32 m
1912	7.92 m
1930	9.14 m
1946	9.75 m
1950	10.36 m
1970	11.58 m
2000–2005	12.80 m

In this study, we aimed to improve understanding of how increasing salinity caused by RSLR and amplified by river dredging affect mature bald cypress growth patterns and climate‐growth relationships in Smith Creek, a tributary of the Cape Fear River, NC. In a novel approach, we combine the use of two proxies to accomplish this—foraminifera as a saltwater indicator and tree rings to assess growth suppression in bald cypress.

### 1.1 Microfossils as a Saltwater Indicator

Foraminifera, a type of single‐celled microorganism with agglutinated or calcareous tests that live only in brackish to saline environments, can be used as a proxy for chronic salinity changes (Edwards and Horton 2000; Horton and Culver 2008). As live organisms responding to environmental conditions, their presence indicates the onset and continued salinization of a given environment. While foraminifera have primarily been employed as a proxy for sea level, some studies have also evaluated the impact of salinity on foraminiferal distribution (e.g., Williams 1995; de Rijk and Troelstra 1997; Verlaak and Collins 2021). The distribution of different species of benthic agglutinated foraminifera found in marsh sediment is controlled by the frequency and duration of tidal inundation, and salinity tolerance in foraminifera is a function of this flooding/exposure tolerance (e.g., Abbene et al. 2006; Kemp, Horton, and Culver 2009; Kemp, Horton, Corbett, et al. 2009; Kemp et al. 2017). It is therefore possible to determine past salinity conditions, as well as shifts to new salinity regimes, by quantifying the abundance and species of foraminifera in a sediment profile. The timing of different salinity conditions can then be determined through age dating techniques.

While foraminifera can be used as a saltwater indicator, thecamoebians, a type of unicellular testate amoeba, can be used as a freshwater indicator (Patterson and Kumar 2002; Barnett et al. 2017). A small number of thecamoebians have been found to tolerate brackish conditions, but they are generally associated with freshwater environments (Charman et al. 1998). Paired with a lack of foraminifera, the presence of thecamoebians in a sediment profile may therefore indicate freshwater conditions during the period in which that layer of sediment was deposited. Radioactive dating techniques, such as ^137^Cs pollution dating, can be used to estimate the approximate year during which salinity began to increase in sediment cores by dating the portion of the core where foraminiferal assemblages appear and thecamoebians decrease or disappear.

### 1.2 Bald Cypress and Dendrochronology

Bald cypress wetlands include some of the oldest trees along the coast of the southeastern US, with recorded ages exceeding 2000 years (Stahle et al. 2019). As a freshwater species that withstands flooding, bald cypress dominate most freshwater swamps in this region. Bald cypress occurs in a wide range of environments, including tidal swamps near coasts and nontidal wetlands such as those along blackwater streams (Stahle et al. 2012). Bald cypress are highly tolerant of disturbance events such as hurricanes due to their buttressed bases and intricate root systems that allow them to endure wind damage and occasional saltwater flooding (Conner 1995).

Although bald cypress is generally considered a freshwater species, it is somewhat salt tolerant. Among tree species studied for their response to increased salinity, bald cypress exhibits the greatest degree of salt tolerance (e.g., Conner et al. 2007; Powell et al. 2016; Stahle et al. 2012; Stotts et al. 2021). For example, studies have demonstrated 100% mortality in swamp chestnut oak and water oak compared to 100% survival of bald cypresses when experimentally flooded for 11 weeks with low levels of salinity (2 ppt; Conner 1994; Conner 1995; Conner et al. 1998; McLeod et al. 1996). It is therefore understood that bald cypress can tolerate low levels of salinity over prolonged periods that are sometimes present in tidal creeks as well as episodic events of higher salinity associated with storm flooding or droughts (Conner and Askew 1992; Conner et al. 2007; Elcan and Pezeshki 2002; McLeod et al. 1996; Pezeshki 1990). However, most previous studies on the saltwater tolerance of bald cypress were conducted on seedlings or saplings in controlled environments. Fewer studies have investigated the impacts of salinity in situ on mature bald cypress trees. However, evidence demonstrates that while bald cypress can tolerate low levels of salinity (< 5 ppt), tree stands are less dense, less diverse, and have narrower tree rings than those growing in freshwater conditions (Yanosky et al. 1995; Thomas et al. 2015). Additionally, the boundaries of bald cypress ghost forests along the Neuse River, NC, have been found to co‐occur with boundaries of elevated salinity caused by RSLR, suggesting prolonged salinity exposure leads to tree mortality (Phillips 2024). However, additional research to determine the threshold of salinity tolerance and the ability of mature bald cypress trees to recover from saline flooding is needed.

Dendrochronology, the study of tree rings (Speer 2010), has been used extensively to study bald cypress, primarily in non‐tidal settings (e.g., Stahle et al. 1988; Stahle and Cleveland 1992; Stahle et al. 2011; Stahle et al. 2012; Thomas et al. 2015; Stahle et al. 2019; Tucker et al. 2022). Unfavorable growth conditions produce narrower annual rings which, when analyzed in a tree‐ring chronology, can be compared to instrumental records of past conditions (e.g., temperature, precipitation, drought, or flooding) to determine the role of these variables in limiting tree growth. A tree‐ring chronology is a site‐level representation of tree growth that can be used to examine shared signals that result from ecological or climatic conditions that impact tree stands (Speer 2010).

Bald cypress chronologies have been found to be sensitive to precipitation because of the close relationship between precipitation and water levels, dissolved oxygen, and moisture and nutrient flux to the roots (Stahle et al. 2012). Drought conditions measured with the Palmer Drought Severity Index (PDSI) can also impact water levels, dissolved oxygen, and nutrient delivery in a swamp and may even be a more comprehensive measurement of water conditions in these types of environments since PDSI considers both water input and output (Haddinghaus and Sabol 1991). Additionally, drought conditions can further affect these environments by increasing salinity (Jones and van Vliet 2018). Chronically elevated salinity levels have been found to reduce water use in bald cypress, which can limit radial growth (Krauss et al. 2009; Krauss and Duberstein 2010). Therefore, while precipitation is generally thought to be the climate variable that bald cypress are most sensitive to, PDSI may have a greater control on radial growth for bald cypress growing in tidally influenced habitats where salinity is an important factor in addition to water levels. Short‐term elevated salinity caused by storm surge during hurricanes, for example, can also cause stress in bald cypress chronologies. Especially for seedlings, hurricanes cause reduced radial growth and survival in bald cypress (Conner and Askew 1992). Additionally, false ring formation in bald cypress is more likely to coincide with years with high tropical cyclone activity (Tucker et al. 2022).

Since bald cypress is one of the dominant species in forested freshwater tidal wetlands, a better understanding of the effects of salinity on bald cypress is important for understanding the ways in which much of the southeastern US coastal landscape will continue to change as global and relative sea levels continue to rise. In addition to providing important habitat for a variety of species, including migratory birds and waterfowl, bald cypress provide valuable ecosystem services. These include mitigating flooding during storm events, improving water quality, controlling pollution and sediment input, and storing carbon (Acreman and Holden 2013; Field et al. 1991). In this study, we aimed to develop a better understanding of the process of ghost forest formation to characterize changes to ecosystem structure and ecosystem services that may occur as RSLR continues. We used dendrochronology and sediment analyses of foraminifera and thecamoebians to test the following hypotheses:

Hypothesis 1 *Bald cypress will exhibit signs of suppressed growth as salinity increases following river dredging events and associated saltwater intrusions*.

Hypothesis 2 *Foraminifera will become more abundant in shallower (younger) layers of sediment cores (and thecamoebians less abundant), which will correspond temporally with periods of saltwater‐induced stress in bald cypress chronologies*.

## 2 Methods

### 2.1 Site Selection

Smith Creek is a tidal creek located in the north‐central portion of New Hanover County in southeastern North Carolina, USA. It is a tributary of the Cape Fear River, and drains a watershed that spans > 67 km^2^ (16,650 acres; Rosov 2020). The Wilmington tide gauge, operated by NOAA and located ~5 km southwest from the mouth of Smith Creek, has recorded a RSLR of ~0.26 m (0.86 ft) since its installation in 1935 at a rate of ~2.73 mm/year (NOAA 2023). Tidal range and storm surges in Wilmington have been exacerbated by dredging of the Cape Fear River (Familkhalili and Talke 2016).

**FIGURE 1:**
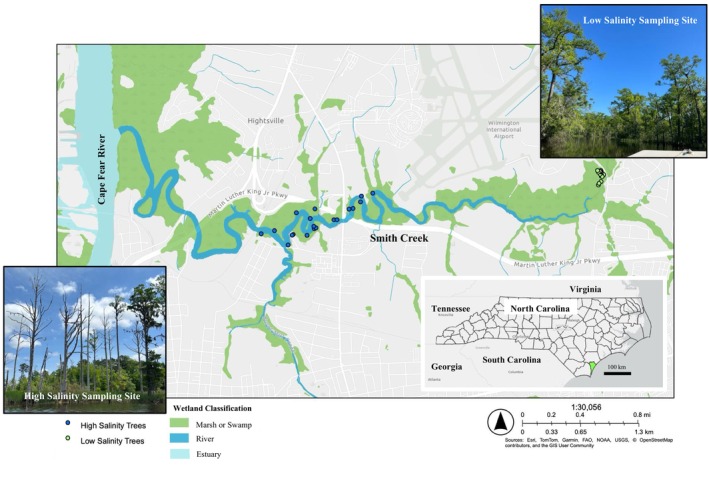
Map of the study area. Smith Creek and the Cape Fear River are labeled, and green shading denotes the locations of marshes and swamps surrounding these waterways. An inset map shows the state of North Carolina with New Hanover County shaded in green. Images and locations of the high‐ and low‐salinity sampling sites are included.

Smith Creek is lined by forested freshwater tidal wetlands dominated by bald cypress (Figure 1). A visual assessment indicates that ghost forests are present in marshes along the lower portions of Smith Creek, but the forests at the upper portions of Smith Creek lack significant mortality (Figure 1). Dendrochronological analyses of tree growth coupled with sediment analyses are used here to verify that elevated salinity happens concurrently with suppressed bald cypress growth and to help estimate when the ghost forests first formed.

Two bald cypress sampling sites were selected along the length of Smith Creek to capture a salinity gradient. A low‐salinity sampling site (< 5 ppt) was selected near the head of Smith Creek, and a high salinity sampling site (8–12 ppt) was selected nearer to the mouth of Smith Creek, where it flows into the Cape Fear River (Figure 1). We used a handheld SonTek CastAway CTD (conductivity, temperature, and depth) sensor to measure salinity conditions at 12 stations along the length of the creek during multiple trips to Smith Creek over 18 months. Our two sites, which represent both low salinity and higher salinity, allow for analyses of bald cypress growth under different salinity ranges.

### 2.2 Microfossil Analyses

Our sediment analysis included two phases. First, to examine whether the occurrence of foraminifera and thecamoebians (hereafter, “microfossils”) is consistent with the current salinity gradient along Smith Creek, a sediment volume of 2 cm^3^ from the top layer (1 cm) of sediment was sampled at 12 stations spaced along the ~10 km length of the creek. A real‐time kinematic (RTK) GPS unit was used to record the latitude, longitude, and elevation of coring sites. A modern microfossil analysis was then completed at each station to determine whether foraminifera and thecamoebians are present with modern salinity conditions. Each sample was wet sieved at 500 and 32 μm and the sediment from the 500–32 μm fraction was analyzed under a dissecting microscope to pick, identify to the species level, and count microfossils (Kemp, Horton, Corbett, et al. 2009).

Second, to determine when salinity first started increasing in Smith Creek and to track changes in salinity over time, a peat corer was used to collect three 100‐cm‐long sediment cores at the high salinity sampling site and one 100‐cm‐long sediment core from the low‐salinity sampling site. For microfossil analyses, cores were sub‐sampled at 5‐cm intervals by taking 2 cm^3^ of sediment from the core at each depth interval. This sediment was analyzed in the same manner as in the modern analysis. Relative microfossil abundance (%) from the bottom to the top of the core was used to track changes in abundance and species over time.

The two additional sediment cores collected at the high salinity sampling site were sent to East Carolina University to develop a ^137^Cs chronology. ^137^Cs (half‐life 30 years) is an artificially produced radionuclide present in the environment due to atmospheric fallout from nuclear weapons testing, reactor accidents, and discharges from nuclear facilities. Global dispersion and fallout of ^137^Cs began in 1954 following the detonation of high‐yield thermonuclear weapons with a distinct maximum in fallout in 1963 (Pennington et al. 1973). Cores were sectioned into 1 cm intervals, and each interval was analyzed for ^137^Cs activity by gamma spectroscopy (662 keV) on low‐background, high‐efficiency, high‐purity Germanium detectors. A corresponding ^137^Cs peak activity in a sediment core provides a time horizon for the 1963 sediment layer (Young et al. 1995).

### 2.3 Dendrochronology

We sampled bald cypress at both the low‐ and high‐salinity sites. We used a targeted sampling approach to favor older trees in our dendrochronological analysis because we wanted our record of tree growth to extend for a century or more to capture changes associated with dredging of the Cape Fear River. At the low‐salinity site, 17 live bald cypress trees were selected for sampling. At the high‐salinity site, 15 live bald cypress trees and six snags (standing dead trees) were selected. For the live trees, a 12.7‐mm‐diameter increment borer was used to extract two cores from opposite sides of each tree at a minimum of one meter above any buttressing of tree bases. Coring height and diameter at coring height of each tree were measured. A chainsaw was used to extract a full or partial cross‐section from the six snags, and diameter at breast height was recorded. An RTK GPS unit was used to record the latitude, longitude, and elevation of each sampled tree.

In the laboratory, we followed standard dendrochronological methods to process and date our cores (Speer 2010). Cores were air dried before being mounted on pieces of wood and sanded with progressively finer sandpaper (80, 120, 220, 320, and 400 grit sandpapers) to improve the visibility of the tree rings. Sanded tree cores were then scanned to images at 1200 dpi using a flatbed scanner. CooRecorder and CDendro programs were used for tree‐ring analysis (Maxwell and Larsson 2021). We counted and measured the tree rings in each core. Tree rings were then visually crossdated using the list method for identifying marker rings among samples (Speer 2010). COFECHA software was used to statistically verify the visual crossdating results (Holmes 1983). This allowed us to date the tree rings to their approximate year of formation and develop tree‐ring chronologies (Speer 2010). Ring‐width series in both chronologies were truncated at the year 1850 to focus on the period of interest and to optimize shared signals. Before 1850, series were not crossdated, but rings were carefully counted to obtain approximate tree ages.

### 2.4 Statistical Analyses

We assessed the quality of our chronologies using descriptive statistics including series intercorrelation value, signal‐to‐noise ratio, and expressed population signal through the dendrochronology program library (*dplR*) in R version 4.3.1 (R Core Team 2023). The average age of trees in each sampling site was compared using the non‐parametric Mann–Whitney *U* test of difference. The chronologies were standardized in *dplR* using a negative exponential curve to account for the decrease in ring widths that occurs as trees age.

The R package *treeclim* was used to assess and compare climate‐growth relationships present in each standardized chronology (Zang and Biondi 2015). Pearson's linear correlation estimates were used to assess relationships between monthly precipitation, PDSI, and average temperature from 1895 to 2022 (NOAA National Centers for Environmental Information Divisional Time Series). Each year's climatic variables for the months of January–October were compared to that year's standardized ring widths. The previous year's climatic variables for the months May–December were compared to the current year's standardized ring widths.

To examine the history of stress in the bald cypress trees, we used two approaches. First, we constructed a suppression chronology for each site to determine individual years of suppressed growth shared among the trees. A 20‐year running mean of raw ring widths was first calculated, then each year's ring widths for each tree were compared to this running mean. If ring width was less than 25% of the 20‐year running mean, we considered that year's growth for that tree as “suppressed.” Raw ring widths were used because a standardized chronology has the potential to remove ecological signals in the ring widths that would indicate suppression. When suppressed growth occurred in over 75% of trees, that year was considered to have “widespread suppression.” These parameters were modeled after the methods presented in Tucker et al. (2018). To combine the suppression analysis with the climate analysis, we used the climate variable found to have the strongest relationship to tree growth, and then examined whether years of suppression were characterized by similar climate conditions.

We also aimed to identify longer periods of suppression (extending two or more years) at the high salinity site, when compared to the low‐salinity site using the program *dfoliatR* (Guiterman et al. 2020). The *dfoliatR* program was initially designed to examine the effects of forest pests on tree growth but can be used for other applications. *dfoliatR* uses a standardized tree‐ring chronology from a host species (i.e., the species impacted by stress) and a standardized tree‐ring chronology from a non‐host species that is unaffected. In this study, we compare our high‐salinity chronology (“host”) and our low‐salinity chronology (“non‐host”) to examine the suppression signal resulting from high salinity levels. We identified longer periods of suppression events affecting the majority of trees at the high salinity site, and we determined the level of severity of these periods of suppression.

## 3 Results

### 3.1 Microfossil Analyses

**FIGURE 2:**
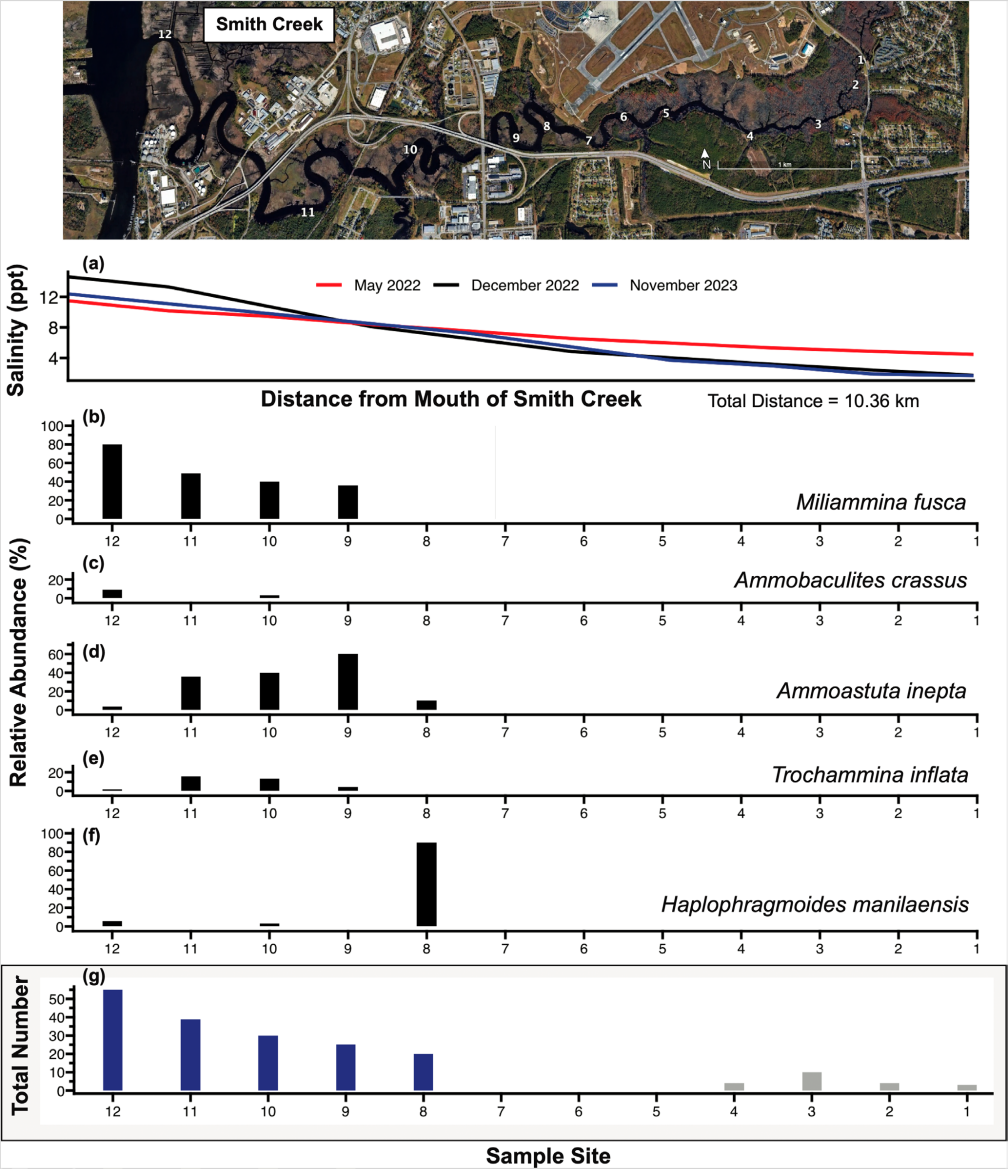
Analysis of modern foraminiferal assemblage in Smith Creek. Twelve surface sediment samples were collected at the locations on the map, and the species and number of foraminifera were recorded. (A) a salinity gradient along the ~10.36 km length of Smith Creek, as recorded on three separate days over the course of 18 months. (B)–(F) Relative abundance of species present in each sample, and (G) the total number of foraminifera (blue) and thecamoebians (gray) present in each surface sediment sample.

The foraminifera and thecamoebians found in the 12 stations from the modern analysis confirm the presence of a salinity gradient (Figure 2A). Thecamoebians dominate stations 1–4 in the upper half of Smith Creek where salinity ranges from 0 to 5 ppt (Figure 2G). A statistically relevant number of foraminifera (minimum of 20 individuals) began to appear at Station 8, with the greatest number of foraminifera found in Sample 12 (55 individuals), which is located at the mouth of Smith Creek and has the highest salinity (up to 12–14 ppt; Figure 2G). The species of foraminifera found in the modern stations in Smith Creek include *Miliammina fusca* , *Trochammina inflata* , *Ammoastuta inepta* , *Ammobaculites crassus* , and *Haplophragmoides maniliaensis*. In the samples collected at the highest salinity stations (Stations 11 and 12), *M. fusca* was the most common species (19 individuals; Figure 2B). *A. inepta* (15 individuals in Station 9) and *H. manilaensis* (18 individuals in Station 8) were more abundant farther upstream where salinity is lower (Figure 1D–F; see Appendix S1 for raw counts of microfossils in all samples).

**FIGURE 3:**
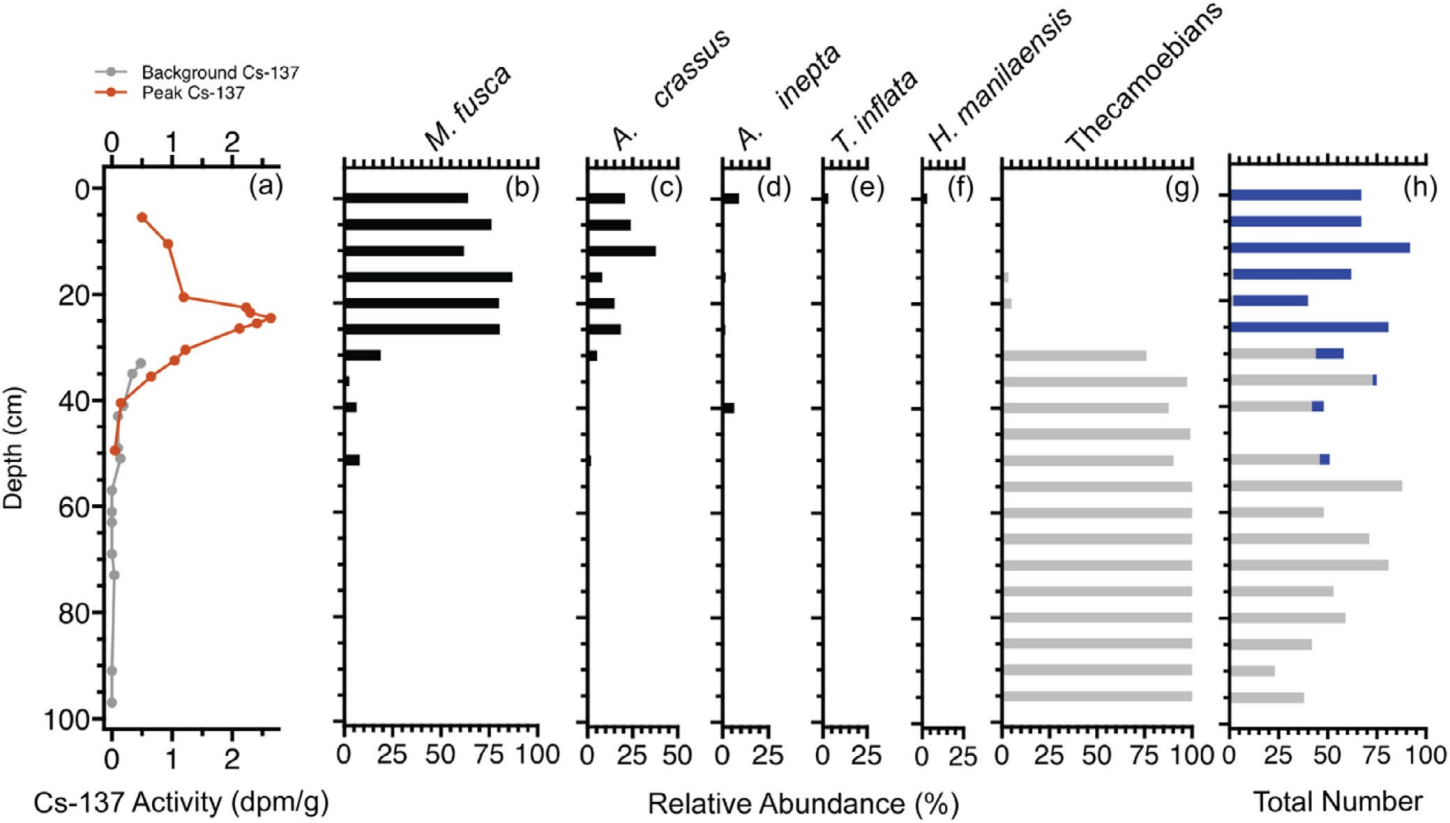
High salinity sampling site (A) ^137^Cs activity and (B–H) microfossil analysis. Sediments were analyzed at every 5 cm of the core. Five species of foraminifera ( *Miliammina fusca* , *Amobaculites crassus*, *Ammoastuta inepta* , *Trochamina inflata*, and *Haplophragmoides manilaensis* ) were found. Thecamoebians (gray) began appearing at 15 cm, and foraminifera were not found below 50 cm. The peak of ^137^Cs activity, corresponding with 1963, occurs around 25 cm.

The high salinity sediment core was devoid of foraminifera from 100 to 50 cm of depth. Foraminiferal abundance generally increased toward shallower depths of the core, beginning with six individuals at 40 cm (Figure 3). The high salinity sediment core had the greatest abundance of foraminifera in the topmost layers of sediment (e.g., 67 individuals at both 0 and 5 cm; Figure 3). Thecamoebians dominate the core from 100 to 30 cm of depth, but they decrease in abundance above 30 cm (see Appendix S1 for raw counts). In the low‐salinity sediment core, no foraminifera were found at any depth from 50 to 0 cm (see Appendix S1) and thecamoebians were present at all depths. ^137^Cs activity peaked at around 25 cm depth in the high salinity site core, which corresponds with 1963 (Pennington et al. 1973).

### 3.2 Dendrochronological Analyses

The tree‐ring chronologies for the low and high salinity sampling sites included a similar number of series and had similar values for descriptive statistics (Table 2). The low‐salinity sampling site chronology included 22 series from 12 of the 17 sampled trees with a series intercorrelation value of 0.503 (Table 2; see Appendix S1). The high salinity sampling site chronology included 25 series from 17 of the 21 sampled trees with a series intercorrelation value of 0.416 (Table 2; see Appendix S1). Some series were left out due to challenges in crossdating that could not be resolved. Ring‐width series in both chronologies were truncated at the year 1850 to focus on the period of interest over which dredging occurred and to optimize shared signals and sample depth. Five out of the six sampled snags from the high‐salinity site were included in the high‐salinity chronology. The outer rings of the six snags were dated to 1941 (SMC120; not included in the chronology), 1953 (SMC117), 1964 (SMC116), 1988 (SMC111), 1994 (SMC118), and 2005 (SMC112). We expect that these trees died later than the outer rings indicate because all snags had significant signs of decay, including the loss of wood and the absence of bark.

**TABLE 2:** Dendrochronological statistics for the high‐salinity and low‐salinity sampling site chronologies.

	High‐salinity chronology	Low‐salinity chronology
Number of series	25	22
Series intercorrelation	0.416	0.503
Average mean sensitivity	0.377	0.382
Expressed population signal	0.901	0.911
Signal‐to‐noise ratio	9.081	10.264

**FIGURE 4:**
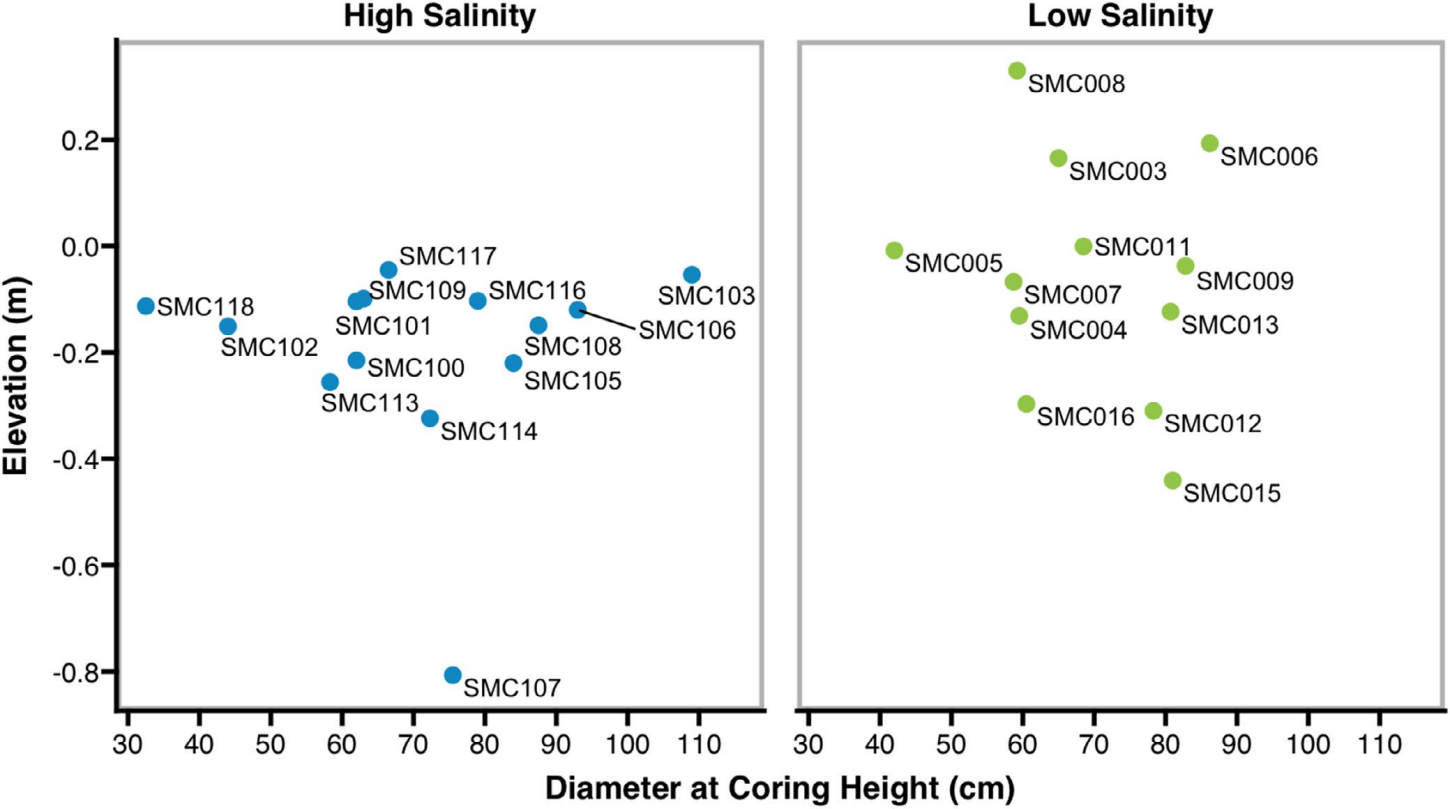
Elevations (m) relative to MHW and tree diameter (cm) at coring height of trees included in the high‐ and low‐salinity chronologies. Neither elevations (*p* = 0.0746) nor diameter (*p* = 0.753) were significantly different between the two sampling sites.

We found no significant differences in tree elevation and tree diameter at coring height among the two sites (Figure 4). Elevations of trees were not normally distributed, so the non‐parametric Mann–Whitney *U* test of difference was used to compare elevations between the two sites. Most trees (88%) that were included in the chronologies were growing below mean high water (MHW; Figure 4). The median elevation of all trees sampled was −0.120 m below MHW. The diameter at coring height for each sampling site was normally distributed, so a *t*‐test was used to compare diameters. The mean diameter at coring height of all trees sampled was 69.7 cm. Two trees were left out of the high salinity dataset when comparing elevations of sampled trees due to no elevation data, and one snag was left out when comparing diameters at coring height of sampled trees due to no diameter measurement.

**FIGURE 5:**
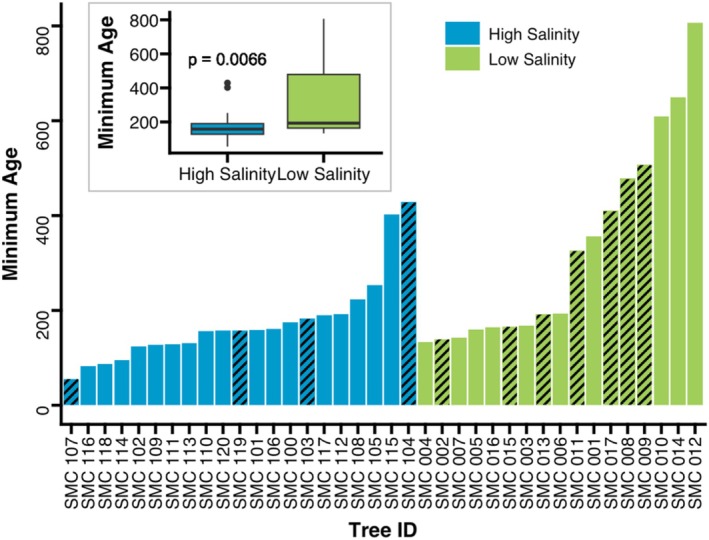
Minimum age of each bald cypress tree sampled grouped by sample site. Diagonal lines indicate which cores did not reach near pith, indicating that these trees may be older than represented here. The inset box plot compares the average minimum ages of trees at each site. The *p*‐value of 0.0066 indicates that the difference between the ages of trees at each site is statistically significant with the low‐salinity site having older trees.

Minimum tree ages differed between the two sites, with trees in the low‐salinity site being older than those in the high‐salinity site (Figure 5). A non‐parametric Mann–Whitney *U* test of difference indicated that tree ages differed among the two sites; median age at the high‐salinity site was 158 years, whereas the median age at the low‐salinity site was 193 years, and these ages were statistically different (*p* = 0.0066). The trees we sampled at the high‐salinity site were relatively even‐aged, whereas age varied across a wider gradient at the low‐salinity site. Additionally, given that some of our tree cores did not reach the innermost ring (pith) of the tree (29% of cores did not reach pith), especially in the low‐salinity sampling site, and cores were collected above ground level, sampled trees are slightly older than reported here. Another Mann–Whitney *U* test of difference between only the tree cores that did reach pith indicated that absolute tree age also differed among the two sites (*p* = 0.0020). Minimum tree age as reported here refers to the innermost ring observed in our cores at coring height.

**FIGURE 6:**
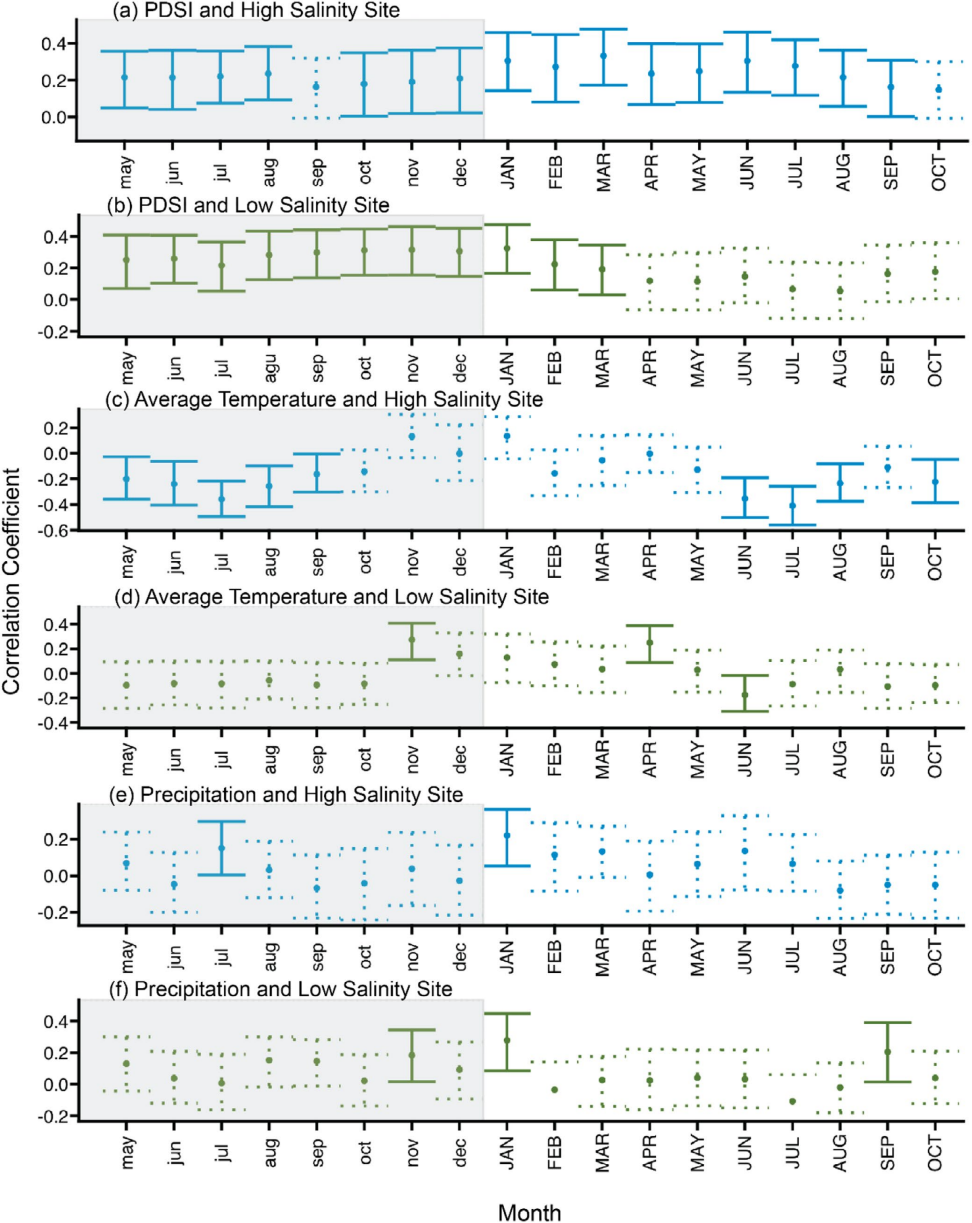
Static linear correlations between tree growth (as measured with standardized tree‐ring widths) at each sampling site and (A, B) PDSI; (C, D) average temperature; and (E, F) precipitation. Dashed lines indicate non‐significant correlations and solid lines indicate significant correlations (*p* < 0.05). Months from the previous year are written in lowercase letters and the plot is shaded in gray, and months from the current year are written in uppercase letters.

The R package *treeclim* was used to determine climate‐growth relationships using each chronology, and the analysis indicated that the high salinity sampling site was more sensitive to climatic variability than the low‐salinity sampling site (Figure 6; see Appendix S1 for climate records). PDSI had the most consistent influence on tree growth. Growth in the high salinity sampling site was positively correlated with PDSI for every month except for September of the previous year and for October of the current year (*p* < 0.05; Figure 6A). Significant positive correlations between PDSI and growth in the low‐salinity sampling site predominantly occurred with the previous year's PDSI (Figure 6B). This indicates that drought periods correlated with narrower tree rings and moist periods correlated with wider tree rings from 1895 to 2022. The climatic analysis indicates that average temperature also has more significant correlations with the high‐salinity chronology than the low‐salinity chronology (Figure 6C,D). These correlations are negative, indicating that higher temperatures correlate with narrower rings. Precipitation did not have a strong influence on tree growth in either chronology (Figure 6E,F).

**FIGURE 7:**
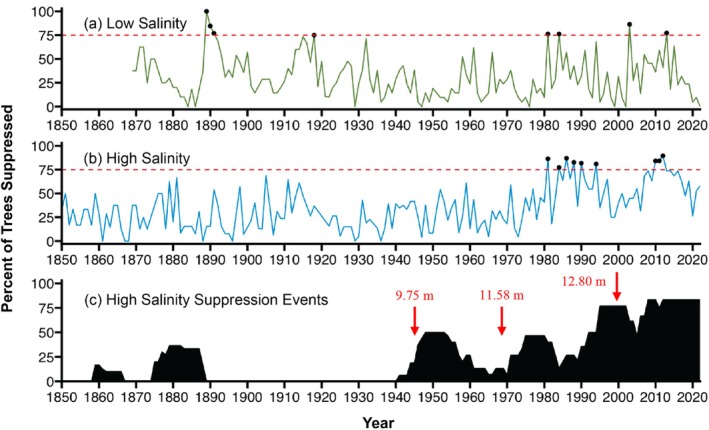
(A) Percent of trees for each year that had suppressed growth in the low‐salinity site and (B) percent of trees for each year that had suppressed growth in the high‐salinity site. A red dashed line at 75% indicates the threshold for widespread suppression. Black points on the lines highlight years with widespread suppression. (C) Five suppression events occurred in the high salinity sampling site over the study period: 1859–1860, 1877–1887, 1946–1960, 1971–1983, and 1985–2022. The percent of trees involved in each suppression event is shown in plot (C). Suppression events were identified by comparing the high salinity sampling site chronology to the low‐salinity sampling site chronology, so only the events in the high salinity sampling site are shown. Years and depths of major dredging events in the Cape Fear River are denoted with red arrows in plot (C).

To determine differences in tree growth between the high‐ and low‐salinity sampling sites, suppression chronologies were built for each site and individual years with widespread suppression (suppressed growth in > 75% of trees) during the period of analysis (1850–2022) (Figure 7). The high salinity sampling site had 9 years with widespread suppression, whereas the low‐salinity sampling site had 8 years with widespread suppression. In the high‐salinity sampling site, widespread suppression occurred exclusively in the last several decades (Figure 7B and Table 3) and nearly every year (except for 1984) of widespread suppression was associated with a negative PDSI value corresponding to drought conditions (Table 3). Widespread suppression in the low‐salinity sampling site did not have this same temporal trend and was not frequently associated with negative PDSI values, although PDSI data were unavailable for three of the seven suppression years (Figure 7A and Table 3).

**TABLE 3:** Annual PDSI values for years with widespread suppression for the high‐ and low‐salinity sampling sites (NOAA Divisional Time Series Portal).

	Years with suppression	PDSI value
High‐salinity sampling site	1981	−0.873
	1984	0.840
	1986	−2.404
	1988	−1.210
	1990	−1.760
	1994	−0.360
	2010	−0.863
	2011	−2.834
	2012	−2.617
Low‐salinity sampling site	1889	Unavailable
	1890	Unavailable
	1891	Unavailable
	1918	−0.579
	1984	0.840
	2003	1.649
	2013	0.834

In addition to examining individual suppression years at each site, we also looked at more extended suppression events in the high‐salinity site relative to the low‐salinity site using the *dfoliatR* package in R. This method uses the low‐salinity sampling site chronology as a baseline to compare against the high salinity sampling site chronology. There were five suppression events identified over the study period (1850–2022): 1859–1860, 1877–1887, 1946–1960, 1971–1983, and 1985–2022 (Figure 7C). The most recent suppression event from 1985 to 2022 was the longest event and included the greatest number of trees impacted (~90% of trees from 2008 to 2022).

Examining the severity of suppression in each individual tree of the high‐salinity site using the *dfoliatR* package indicated that most periods of suppression occurred after 1950, and most moderate and severe suppression occurred after 1980. These periods were identified by comparing individual tree series from the high salinity sampling site to the chronology from the low‐salinity sampling site. When grouped together, these individual periods of suppression form the suppression events identified in Figure 7C.

## 4 Discussion

We found that tree‐ring growth suppression and increased sensitivity to climate variability are associated with increasing saltwater intrusion over the last century. Our microfossil analysis confirms that salinity has been elevated in the lower portion of Smith Creek (the high salinity site) since the early 1900s. In contrast, the upper portion of Smith Creek remains a low‐salinity environment. We observed greater sensitivity of tree growth to PDSI and temperature as well as more frequent and prolonged growth suppression in the high salinity sampling site chronology, suggesting that elevated salinity is the primary driver for these differences. Few studies have focused on the impacts of saltwater intrusion on mature bald cypress trees or on the effects of dredging on forested freshwater tidal wetlands. Therefore, our findings fill gaps in the literature and contribute to greater understandings of how RSLR is expected to continue to impact these wetland communities across much of the southeastern US.

### 4.1 Spatial and Temporal Trends in Microfossil Communities

The type of benthic agglutinated foraminifera observed in the Smith Creek sediment cores only occur in brackish to saline environments, and the different species of foraminifera found are known to live at different levels of salinity (Berkeley et al. 2007). Our modern foraminiferal analysis confirmed that saltwater intrusion has occurred along the sampled length of Smith Creek, resulting in a gradient of higher salinity near the mouth of the creek and lower salinity farther upstream. The greater abundance of *M. fusca* in samples collected closer to the mouth of Smith Creek was expected given *M. fusca* 's tolerance for higher salinities (Figure 2B; Kemp, Horton, and Culver 2009). The greater abundance of *A. inepta* , *T. inflata* , and *H. manilaensis* , which are more tolerant of lower levels of salinity (Kemp, Horton, and Culver 2009), farther up the length of the creek was also expected and aligned with the pattern of decreasing salinity with increasing distance along Smith Creek (Figure 1D–F). The lack of any foraminifera in modern surface sediments from Stations 1–7 (Figure 1G) is consistent with low salinity in the upper portion of Smith Creek. Additionally, Stations 1–4 had a small number of thecamoebians. Most thecamoebians cannot live in saline environments, so the lack of foraminifera along with the presence of thecamoebians indicates that the upper portion of Smith Creek is currently dominated by freshwater conditions (Charman et al. 1998; Patterson and Kumar 2002; Scott et al. 2007). While our instrumental measurements of current salinity levels in Smith Creek demonstrate the conditions in which the bald cypress trees are currently growing at our two sampling sites, the modern foraminiferal analysis provided evidence that these salinity conditions have been ongoing for at least long enough for foraminiferal assemblages to establish and evolve over time.

To determine how long foraminifera have been established in Smith Creek, sediment dating with ^137^Cs was conducted. The peak of ^137^Cs activity, corresponding to 1963, occurred around 25 cm deep in the sediment (Figure 3). Foraminiferal assemblages were established once the sediment at about 30 cm of depth was deposited, suggesting that by the late 1950s, the high salinity sampling site had completely shifted from a forested freshwater tidal wetland to a more brackish environment. Also, foraminifera were more abundant in younger layers of the core, indicating that salinity continued to increase following the initial shift from freshwater to saline conditions that occurred in the 1950s. Most foraminifera were absent from the high salinity sediment core below 30 cm deep, and there were no foraminifera present below 50 cm, at which point the core was dominated by thecamoebians. The lack of foraminifera below 30 cm and the presence of thecamoebians below 50 cm led us to conclude that before the early 1900s (around 40 cm in the core where ^137^Cs activity initially occurs), Smith Creek was a freshwater environment. Dredging events in the Cape Fear River, along with background rates of global RSLR, may have driven this shift from freshwater tidal marsh to salt marsh. Major dredging events occurred in 1912, 1930, 1946, 1950, and 1970, gradually increasing saltwater influence on the Smith Creek tidal marshes.

Our bald cypress chronology demonstrated the most severe suppression at the high‐salinity site during the latter half of the 20th century (discussed in more detail below). The timing of this suppression corresponds with the timing of elevated salinity as indicated by the presence of foraminifera after the late 1950s. While the foraminiferal results cannot provide the same annual temporal resolution that the tree‐ring chronologies can, dating of the sediment cores with ^137^Cs provides evidence that, after the late 1950s, the high salinity sampling site had been converted to a saltwater environment. Comparing the tree‐ring and foraminiferal proxies with one another indicates the importance of rising salinities on changing bald cypress health. Additionally, foraminifera have a higher sensitivity to the saltwater than bald cypress. Foraminifera can be found in an environment that has only slightly increased in salinity (de Rijk and Troelstra 1997). Foraminifera will therefore be present in the environment earlier than a widespread suppression event in the bald cypress trees would occur.

While the high‐salinity site sediment core was dominated by foraminifera above 30 cm, the low‐salinity site sediment core did not contain foraminifera at any depth of the core. It was dominated by thecamoebians at all depths analyzed. Therefore, while saltwater intrusion has been impacting habitats closer to the mouth of Smith Creek for decades, it has not yet noticeably impacted those closer to the head of Smith Creek, based on the microfossil record. Bald cypress at the low‐salinity sampling site do not exhibit signs of stress or suppression like those at the high‐salinity site do, consistent with the microfossil findings. Collectively, our findings from our sediment sampling provided justification to then compare the long‐term effects of higher salinity on bald cypress growth while using the low‐salinity sampling site as a baseline for growth in a similar environment not experiencing salt stress.

### 4.2 Analysis of Bald Cypress Chronologies

Our two bald cypress tree‐ring chronologies are the first for the near coastal region of the Cape Fear River drainage basin of North Carolina, to our knowledge. Both chronologies included a similar number of series, and both were truncated at the year 1850 to focus on the period of dredging in the Cape Fear River. Cores that go back further than this 1850 cutoff were not crossdated, and instead were ring‐counted to provide an estimate of minimum tree age. The trees of both chronologies are growing in permanently flooded and tidally influenced conditions, and they were of similar size and were growing at similar elevations (Figure 4). This indicated that flooding frequency likely did not differ greatly between sites. Crossdating was more difficult in the high‐salinity chronology than in the low‐salinity chronology due to greater variations in ring widths. There was a lower series intercorrelation value for the high salinity sampling site chronology, although both chronologies are characterized by sufficiently strong series intercorrelation values (Speer 2010; Table 2). The signal‐to‐noise ratio in the high‐salinity chronology was also lower than in the low‐salinity chronology (Table 2). Lower signal‐to‐noise ratios are known to result from environmental stressors that cause less uniform growth over time (Speer 2010), so this ratio suggests that the high salinity sampling site trees are growing in more stressful conditions than the low‐salinity sampling site trees.

The comparison between tree ages at each sampling site demonstrated that trees are younger at the high‐salinity site (Figure 5). It has been established that younger mature trees recover more quickly from stress than older mature trees (Au et al. 2022). Therefore, the lack of old trees in the high‐salinity site may indicate that many of the older trees have already been killed due to a reduced ability to recover from stress. Indeed, an abundance of large snags are present throughout the high salinity sampling site. In contrast, lower stress at the low‐salinity site facilitates survival of older trees, and there are few snags in this area of Smith Creek. The abundance of large snags present at the high salinity sampling site coupled with the abundance of centuries‐old live trees at the low‐salinity sampling site also suggests that it is unlikely that Smith Creek was previously logged at high intensity, further suggesting that salinity (rather than another factor such as logging) explains the age difference. Although sampling these snags was of initial interest to us, decay and loss of the outermost rings made tree‐ring analysis of dead trees infeasible.

As saltwater intrusion begins to migrate farther up the length of Smith Creek, it is possible that the older trees found in the low‐salinity site will die before the younger trees. Across the study area (both sites combined), 12 trees were over 200 years old, including one that was over 800 years old. This tree is the oldest documented tree in the Cape Fear region, although older bald cypress trees have been observed in the nearby Pender and Bladen Counties (Stahle et al. 2019). Loss of old‐growth forests can impact ecosystem function and result in the loss of key ecosystem services. Old‐growth forests have unique features including higher biodiversity, greater carbon sequestration potential, and better water flow regulation capabilities (Hilbert and Wiensczyk 2007; Sutherland et al. 2016). Losing older trees will have negative impacts on ecosystem health and could leave nearby communities more vulnerable to extreme events that cause flooding such as hurricanes.

### 4.3 Climate–Growth Relationships

The climate–growth relationships we observed suggest that the high salinity sampling site is more sensitive to drought and temperature than the low‐salinity sampling site, but as discussed below, these results should be interpreted with caution. In contrast to previous studies on nontidal bald cypress (Stahle et al. 2012; Tucker et al. 2022), we did not note any consistent influence of precipitation on tree growth. PDSI was the only variable found to consistently affect both chronologies over our period of analysis (1850–2022). Drought conditions, during which the loss of freshwater through evapotranspiration exceeds freshwater input, may cause both decreased water levels and elevated salinity of tidal streams (Jones and van Vliet 2018). Therefore, droughts may induce greater levels of stress on bald cypress in these environments than either precipitation or temperature variability alone. While growth in both the high‐ and low‐salinity sampling sites had significant correlations with PDSI, only the high salinity sampling site showed consistent correlations with past and current year PDSI (Figure 6A). Similarly, there were a greater number of significant correlations between average temperature and growth in the high salinity site, indicating greater sensitivity to temperature (Figure 6C,D). Previous research has demonstrated that, during extremely hot years, temperature during the summer months (June, July, and August) do correlate with lower growth in bald cypress (e.g., Tucker et al. 2022; Stahle et al. 2012). Our results support this hypothesis because correlations between tree growth in the high‐salinity site and temperature occur mainly in the summer months (Figure 6C,D).

One potential cause for the greater number of significant PDSI and temperature correlations in the high‐salinity site is that elevated salinity has increased stress and therefore made the trees more sensitive to climatic variability. However, these results should be interpreted with caution because tree‐growth relationships can vary over time, with certain variables becoming more or less important (D'Arrigo et al. 2008; Esper et al. 2016; Wilmking et al. 2020). We conducted similar monthly climate correlations for an early and late half of the study period (1895–1960 and 1960–2022), and these results are presented in Appendix S1. This split period analysis indicated that the relationships between tree growth and climate variables do not remain completely stable over time, which could be a result of increased stress caused by elevated salinity in the later period. However, it is well established that even for trees growing in non‐stressful environments, climate relationships can be unstable (e.g., Wilmking et al. 2020). Therefore, there are other potential reasons for the differences in climate responses between the two sample sites and between the two halves of the study period. These other reasons include non‐stationarity in tree response to climate, high rates of plasticity (e.g., in growth rates, cell structure, and physiological processes) that help trees respond to changing environmental conditions over their lifetimes, and changing tree responses as individuals age and grow larger (Wilmking et al. 2020). The non‐stationarity observed in these climate relationships indicates that the changing climate relationships cannot be definitively linked to elevated salinity.

While there is uncertainty in whether our results indicate greater climatic sensitivity as a result of stressful conditions, there is some evidence supporting this interpretation from previous literature. Trees rely on stored carbohydrates to recover from stressful events (e.g., droughts and extreme temperatures), and trees growing in more conducive environments (e.g., freshwater) will have more carbohydrates stored from previous years when no additional stressors occurred (Hartmann and Trumbore 2016; Helm et al. 2023). However, trees growing in permanently stressful environments would have invested more energy into survival rather than storage of carbohydrates even in years where no additional stressors occurred, leading to greater sensitivity to these additional stressors (Jacquet et al. 2014). Other studies that have found increased tree sensitivity to climatic variables following disturbances that elevated stress include Doyle et al. (2021) and Stotts et al. (2021). These results have important implications for the future of these forests as ongoing climate change continues to impact them.

### 4.4 Patterns of Suppression

**FIGURE 8:**
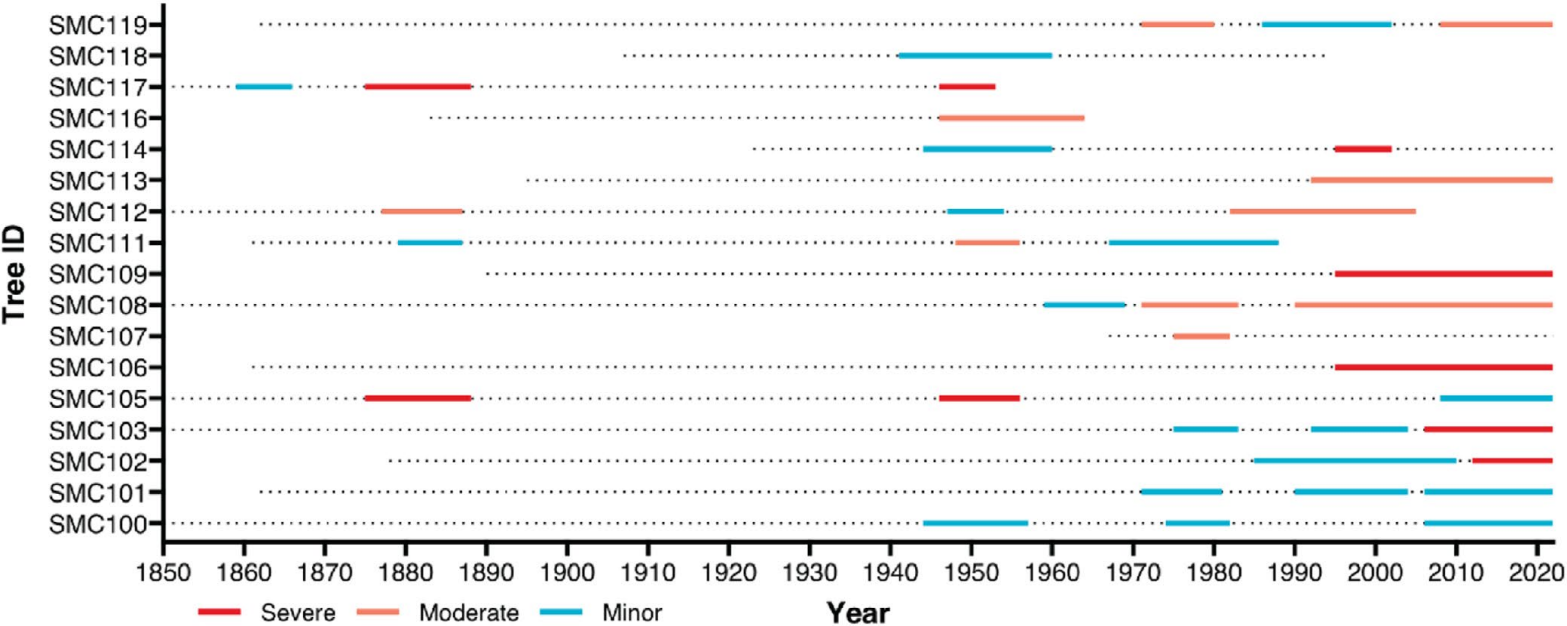
Severe, moderate, and minor suppression periods for each tree sampled at the high salinity site. All three suppression intensities became more common in the later portion of the study period (1950s onward). Periods of suppression were identified using the R package *dfoliatR*.

The suppression analysis indicated that elevated salinity is concurrent with the periods of suppression we observed in the tree‐ring chronology. Analysis with the *dfoliatR* package in R indicated that five suppression events occurred in the high salinity sampling site from 1859 to 1860, 1877 to 1887, 1946 to 1960, 1971 to 1983, and 1985 to 2022 (Figure 7C). Since the low‐salinity sampling site chronology was used as a baseline and was located very close by, most of these suppression events are likely related to differences in salinity rather than climatic events that would have impacted both sites. Three of these periods of stress follow years of known major dredging of the Cape Fear River, which is the main driver for increasing salinity in Smith Creek (Hackney and Yelverton 1990). These major dredging events occurred in: 1946 when the Wilmington Harbor was deepened to 9.75 m (32 ft) below MLLW, 1970 when it was deepened to 11.58 m (38 ft), and 2000 when it was deepened to 12.80 m (42 ft.; Table 1; Leonard et al. 2011). The most recent suppression event (1985–2022) is the longest and continues through the 2000 dredging event (Leonard et al. 2011) and through the present. Additionally, there is only a 1‐year gap (1984) between the two most recent suppression events, suggesting that the trees may have started to enter a permanently stressed state following the 1970 dredging event and that stress was then elevated following the 2000 dredging event. Similar states of permanent stress in bald cypress have been found in other studies (e.g., Thomas et al. 2015; Tucker et al. 2018). The *dfoliatR* package also demonstrated that moderate and severe periods of suppression became more common after ~1980, providing further evidence that stress increased around this time (Figure 8). In contrast, periods of suppression following the 1946 dredging event were mostly minor. These periods of suppression align with when the sediment analysis demonstrated the initial shift to brackish conditions that occurred around the 1950s, but salinities were not elevated enough to cause permanent stress in bald cypress until after the 1970 dredging event.

A recent remote sensing analysis of changes to land cover surrounding Smith Creek (Magolan and Halls 2020) corroborates our findings that the 1970 Cape Fear River dredging event was a likely catalyst for permanent bald cypress stress in the high salinity sampling site. Magolan and Halls (2020) found that the lower portion of Smith Creek, which includes the area where our high salinity sampling site is located, experienced an initial shift from forested wetland to emergent wetland between 1966 and 1980 and increased rates of wetland conversion between 1980 and 2018. These emergent wetlands occur where ghost forests are now found along Smith Creek. In our study, we observed two suppression events around these same time frames (1971–1983 and 1985–2022), suggesting that the saltwater‐induced stress in trees that are still living began at about the same time that Magolan and Halls (2020) observed high tree mortality and the development of ghost forests. Although we are unable to determine exactly when the six snags sampled in our study died due to significant decay and loss of wood containing outer growth rings, they likely died following the start of the conversion from forested wetland to emergent wetland in the 1960s. The results of Magolan and Halls (2020) considered along with our findings provide a better understanding of the timing and extent of change that can occur to forested wetlands because of saltwater intrusion.

While little research has been conducted on saltwater intrusion using mature bald cypress trees, previous studies of bald cypress seedlings have found that continuous inundation with salinities above 10 ppt leads to mortality (Conner 1994). In Smith Creek, our high salinity sampling site has salinities of 8–12 ppt (Figure 2). Since many of our sampled trees are still surviving in these conditions, it appears that some mature bald cypress trees can tolerate salinities above this 10 ppt threshold identified in bald cypress seedlings. However, the presence of ghost forests that began to form from 1966 to 1980 (Magolan and Halls 2020) in the same area as our sampled trees suggests that salinities lower than 8 ppt can potentially lead to mortality in mature bald cypress trees since salinities were likely lower than 8 ppt several decades ago when those trees died. Since younger mature trees have been found to be more robust than older mature trees, and we observed that the surviving trees in the high salinity sampling site are all relatively young (Figure 5), it is possible that younger bald cypress trees can tolerate salinities above 8 ppt whereas older bald cypress trees may die in response to lower levels of salinity. This difference in response to salinity by age as well as natural variation in salinity tolerance among individuals could explain why many trees die in salinities less than 8 ppt while others are still surviving in salinities as high as 12 ppt. As RSLR and dredging of the Cape Fear River continue to facilitate saltwater intrusion into Smith Creek, it is likely that more of the trees in the lower portion of the creek will begin to die. The trees still surviving have consistently narrow rings now, indicating that they are stressed and more vulnerable to mortality events.

## 5 Conclusion

Dredging in the Cape Fear River mimics and exacerbates the effects of RSLR by increasing the amount of water in the estuary, which pushes tides farther upstream and up associated tidal creeks (Hackney and Yelverton 1990). Our findings therefore have applications across broader spatial scales. Specifically, we demonstrated that permanently stressful conditions due to increased salinity could render bald cypress more sensitive to climatic variability. This is likely because bald cypress trees growing in higher salinities focus their energy on survival rather than the storage of carbohydrates (Jacquet et al. 2014); when an additional stressor, such as a drought or hurricanes, occurs, trees are less prepared to withstand these conditions and will experience growth suppression or mortality. Although it was beyond the scope of this study to evaluate correlations between bald cypress stress and hurricanes, it is possible that storm events may have caused some of the stress observed in the chronologies due to the relationship between reduced radial growth and short‐term elevated salinity caused by storm surge (Conner and Askew 1992; Tucker et al. 2022; see Appendix S1 for a list of recent major hurricanes near the study site). It can be expected that as tidal bald cypress forests across the southeastern US are impacted by RSLR, climatic extremes and extreme events will cause more tree mortality than they have in the past due to this increased sensitivity, ultimately leading to the expansion of ghost forests.

As ghost forests form, ecosystem services such as protection from flooding and improved water quality will be lost. Future research should focus on determining the extent of ecosystem services lost as bald cypress forests turn to ghost forests as well as evaluating the impact this will have on surrounding communities where people live. Since bald cypress dominates forested freshwater tidal wetlands in the southeastern US, understanding how communities near these wetlands will be impacted when they convert to ghost forests is important for improving preparedness for extreme events like flooding and hurricanes. For example, storm flooding is expected to increase in both intensity and frequency in the Wilmington, NC area over the next several decades (Kopp et al. 2015). Kopp et al. (2015) determined that, after 2050, the current 1‐in‐10‐year flood is expected to occur in Wilmington almost every year and the current 1‐in‐100‐year flood is expected to occur about every 17–29 years. The loss of ecosystem services as forested freshwater tidal wetlands are converted to emergent wetlands and ghost forests along with this expected increase in storm flooding will make communities more vulnerable to the impacts of flooding.

## Author Contributions

**Kendra Devereux:** data curation (equal), formal analysis (equal), funding acquisition (equal), investigation (equal), methodology (equal), supervision (equal), visualization (equal), writing – original draft (equal), writing – review and editing (equal). **Monica Rother:** conceptualization (equal), funding acquisition (equal), project administration (equal), project administration (equal), resources (equal), resources (equal), software (equal), software (equal), supervision (equal), supervision (equal), writing – review and editing (equal). **Andrea D. Hawkes:** conceptualization (equal), funding acquisition (equal), project administration (equal), resources (equal), software (equal), supervision (equal), writing – review and editing (equal). **Philip Bresnahan:** funding acquisition (equal), resources (equal), validation (equal), writing – review and editing (equal). **D. Reide Corbett:** data curation (equal), resources (equal), software (equal), writing – review and editing (equal). **Roger Shew:** writing – review and editing (supporting).

## Conflicts of Interest

The authors declare no conflicts of interest.

## Supporting information


Appendix S1.

## Data Availability

All data supporting the findings are available within the article and/or its supporting information.
