# Supplementary material for: Tree‐Ring and Sediment Analyses Reveal Processes of Bald Cypress Ghost Forest Formation From Dredging in the Cape Fear River, North Carolina, USA
Source: Ecol Evol. 2025 Aug 28;15(9):e71677. doi: 10.1002/ece3.71677 (PMC12391831; doi:10.1002/ece3.71677)
Supplement: Supplementary file 1 — Appendix S1. [file ECE3-15-e71677-s001.docx]

# APPENDIX A: Microfossils

**Table 1A.** Raw counts of microfossils found in the modern foraminiferal analysis.

| **Sample** | ***Miliammina fusca*** | ***Trochammina inflata*** | ***Ammoastuta inepta*** | ***Haplophragmoides manilaensis*** | ***Ammobaculites crassus*** | **Total Foraminifera** | **Thecamoebians** |
| --- | --- | --- | --- | --- | --- | --- | --- |
| **1** | 0 | 0 | 0 | 0 | 0 | 0 | 3 |
| **2** | 0 | 0 | 0 | 0 | 0 | 0 | 4 |
| **3** | 0 | 0 | 0 | 0 | 0 | 0 | 10 |
| **4** | 0 | 0 | 0 | 0 | 0 | 0 | 4 |
| **5** | 0 | 0 | 0 | 0 | 0 | 0 | 0 |
| **6** | 3 | 0 | 0 | 0 | 0 | 3 | 0 |
| **7** | 5 | 0 | 0 | 0 | 0 | 5 | 0 |
| **8** | 0 | 0 | 2 | 18 | 0 | 20 | 0 |
| **9** | 9 | 1 | 15 | 0 | 0 | 25 | 0 |
| **10** | 12 | 4 | 12 | 1 | 1 | 30 | 0 |
| **11** | 19 | 6 | 14 | 0 | 0 | 39 | 0 |
| **12** | 44 | 1 | 2 | 3 | 5 | 55 | 0 |

**Table 2A.** Raw counts of microfossils found in the high salinity sampling site sediment core.

| **Depth** | ***Miliammina fusca*** | ***Trochammina inflata*** | ***Ammoastuta inepta*** | ***Haplophragmoides manilaensis*** | ***Ammobaculites crassus*** | **Total Foraminifera** | **Total Thecamoebians** |
| --- | --- | --- | --- | --- | --- | --- | --- |
| **0** | 43 | 2 | 6 | 2 | 14 | 67 | 0 |
| **5** | 51 | 0 | 0 | 0 | 16 | 67 | 0 |
| **10** | 57 | 0 | 0 | 0 | 35 | 92 | 0 |
| **15** | 54 | 0 | 1 | 0 | 5 | 60 | 2 |
| **20** | 32 | 0 | 0 | 0 | 6 | 38 | 2 |
| **25** | 65 | 0 | 1 | 0 | 15 | 81 | 0 |
| **30** | 11 | 0 | 0 | 0 | 3 | 14 | 44 |
| **35** | 2 | 0 | 0 | 0 | 0 | 2 | 73 |
| **40** | 3 | 0 | 3 | 0 | 0 | 6 | 42 |
| **45** | 0 | 0 | 0 | 0 | 1 | 1 | 110 |
| **50** | 4 | 0 | 0 | 0 | 1 | 5 | 46 |
| **55** | 0 | 0 | 0 | 0 | 0 | 0 | 88 |
| **60** | 0 | 0 | 0 | 0 | 0 | 0 | 48 |
| **65** | 0 | 0 | 0 | 0 | 0 | 0 | 71 |
| **70** | 0 | 0 | 0 | 0 | 0 | 0 | 81 |
| **75** | 0 | 0 | 0 | 0 | 0 | 0 | 53 |
| **80** | 0 | 0 | 0 | 0 | 0 | 0 | 59 |
| **85** | 0 | 0 | 0 | 0 | 0 | 0 | 42 |
| **90** | 0 | 0 | 0 | 0 | 0 | 0 | 23 |
| **95** | 0 | 0 | 0 | 0 | 0 | 0 | 38 |

**Table 3A.** Raw counts of thecamoebians present in the low salinity sampling site sediment core.

| **Depth** | **Thecamoebians** |
| --- | --- |
| **0** | 7 |
| **10** | 11 |
| **20** | 17 |
| **30** | 2 |
| **40** | 2 |
| **50** | 3 |

# APPENDIX B: Bald Cypress Chronologies


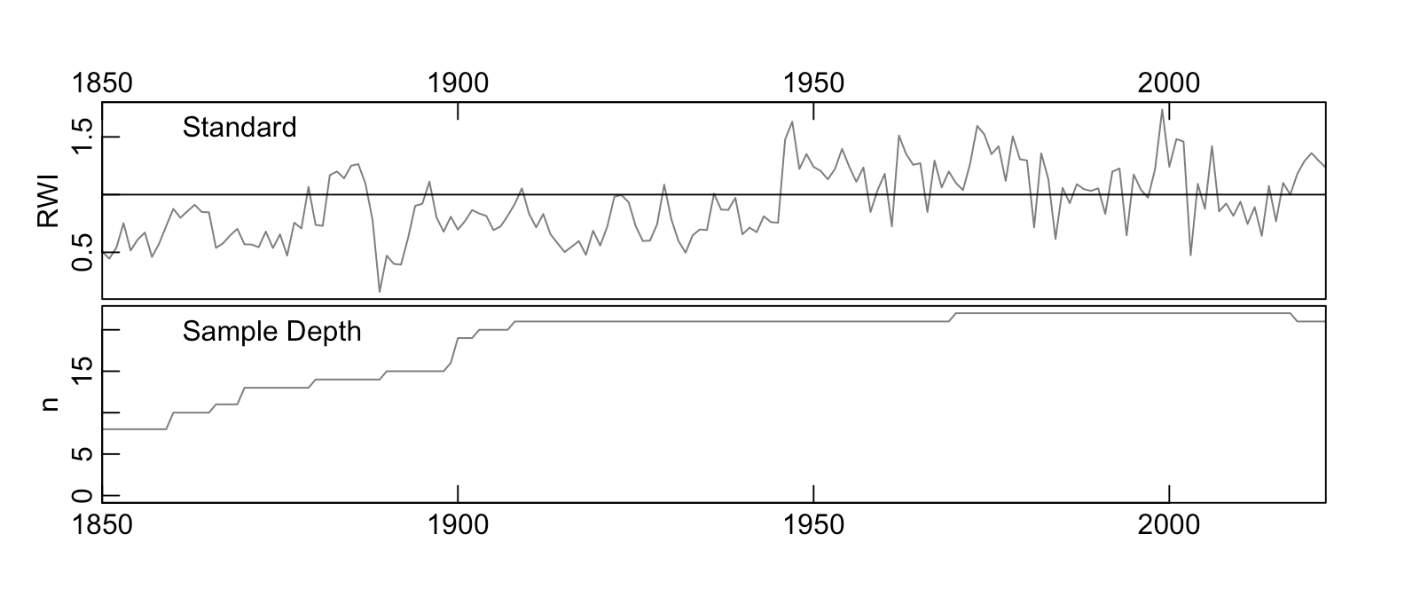


**Figure 1B.** Low salinity sampling site standardized bald cypress chronology. Ring width indices (RWI) are plotted on the top graph and number of samples (Sample Depth) is plotted on the bottom graph.
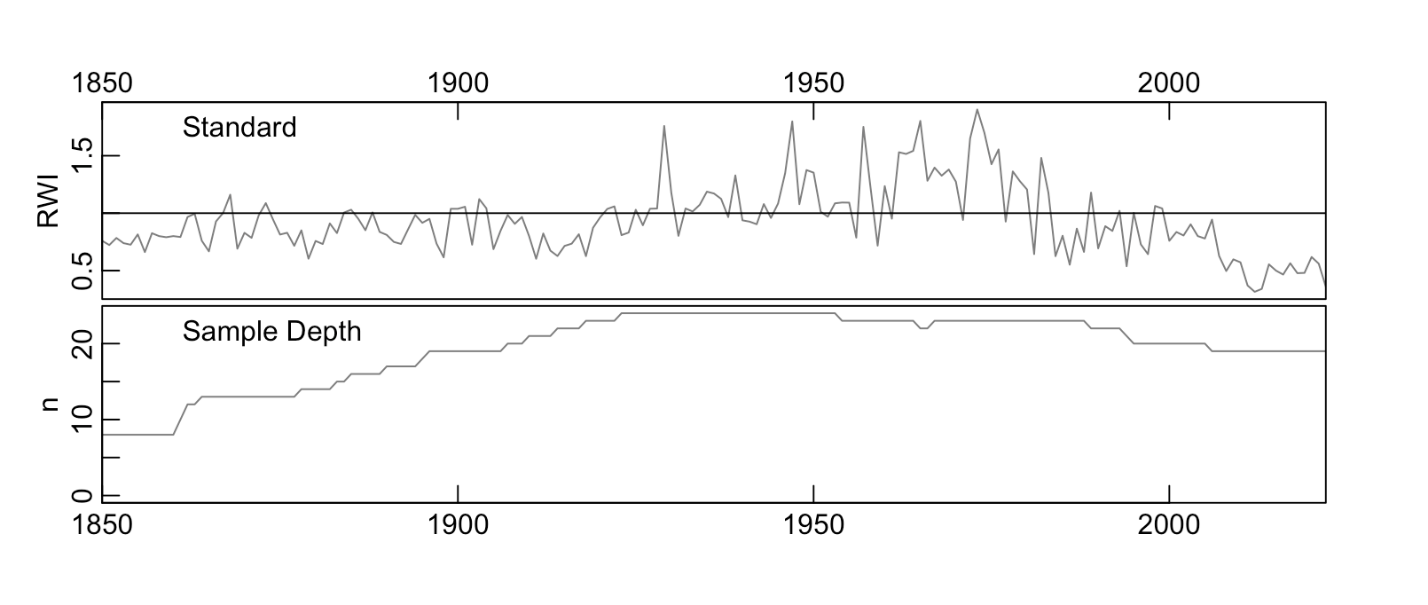


**Figure 2B.** High salinity sampling site standardized bald cypress chronology. Ring width indices (RWI) are plotted on the top graph and number of samples (Sample Depth) is plotted on the bottom graph.

# APPENDIX C: Climate Records and Relationships

**Figure 1C**. Annual precipitation (mm) for southeastern North Carolina. Average annual precipitation is 104.62 mm/yr and there is no significant trend in average annual precipitation over the study period. This precipitation data was downloaded from the NOAA National Centers for Environmental Information (NCEI) Divisional Time Series portal.

**Figure 2C.** Palmer Drought Severity Index (PDSI) for southeastern North Carolina. Negative values indicate drier years and positive values indicate wetter years with greater magnitudes corresponding with more extreme dry/wet years. This PDSI data was downloaded from the NOAA National Centers for Environmental Information (NCEI) Divisional Time Series portal.


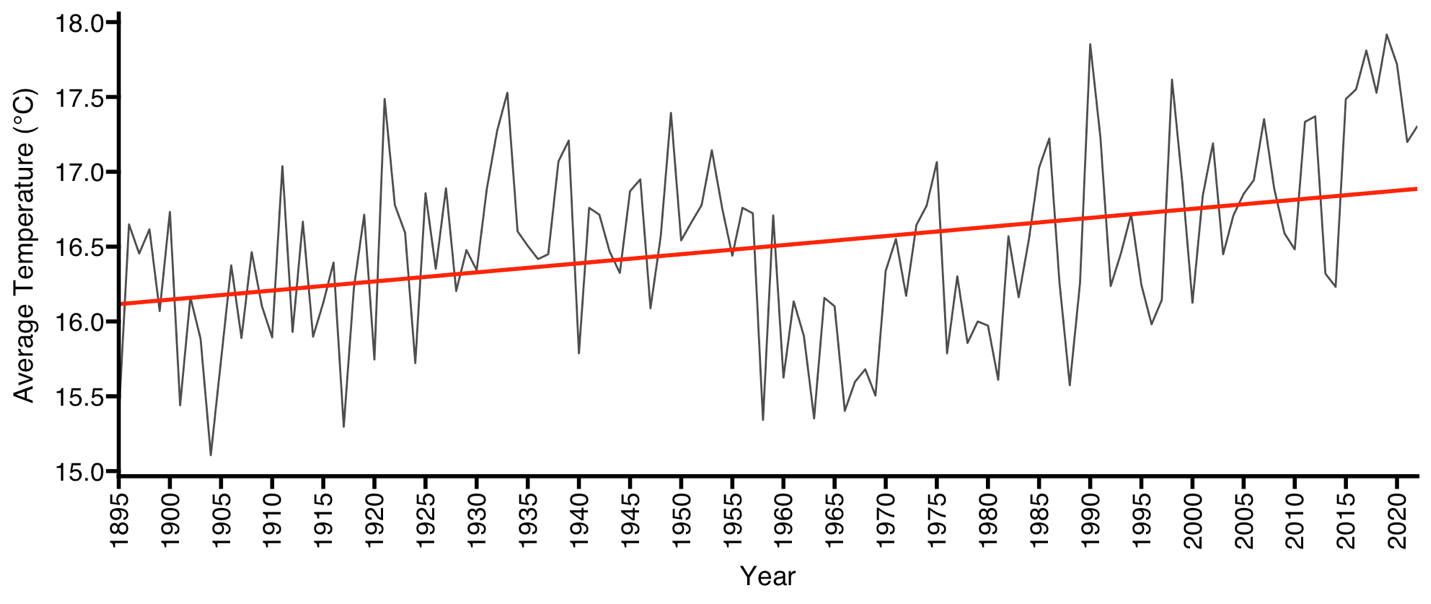


**Figure 3C.** Annual average temperature for southeastern North Carolina. Since 1895, a significant positive trend in average temperature has occurred, with an increase in temperature of approximately 0.774 degrees Celsius. This temperature data was downloaded from the NOAA National Centers for Environmental Information (NCEI) Divisional Time Series portal.

**Figure 4C.** Static linear correlations between tree growth (as measured with standardized tree-ring widths) and PDSI at each sampling site and for two study periods.

**Figure 5C.** Static linear correlations between tree growth (as measured with standardized tree-ring widths) and average temperautre at each sampling site and for two study periods.

**Figure 6C.** Static linear correlations between tree growth (as measured with standardized tree-ring widths) and precipitation at each sampling site and for two study periods.

**Table 1C.** Tropical storms and hurricanes that passed within 25 km of Wilmington, NC from 1922­–2022 (NOAA, 2024).

| **Year** | **Storm Name** | **Storm Category** |
| --- | --- | --- |
| 1946 | Unnamed | 1 |
| 1949 | Unnamed | Tropical Storm |
| 1955 | Diane | 2 |
| 1960 | Brenda | Tropical Storm |
| 1960 | Donna | 4 |
| 1961 | Unnamed | Tropical Storm |
| 1981 | Dennis | 1 |
| 1985 | Kate | 3 |
| 1996 | Bertha | 3 |
| 1996 | Fran | 3 |
| 1998 | Bonnie | 3 |
| 1999 | Floyd | 4 |
| 2002 | Kyle | 1 |
| 2006 | Ernesto | 1 |
| 2012 | Beryl | Tropical Storm |
| 2016 | Hermine | 1 |
| 2017 | Unnamed | Tropical Storm |
| 2018 | Florence | 4 |
